# Unravelling the genomic landscape of Canadian Borrelia burgdorferi: a comparison across global strains

**DOI:** 10.1099/mgen.0.001606

**Published:** 2026-01-08

**Authors:** Anthony Piot, Iain L. Mainprize, Justin Wood, Jeff Gauthier, Cezar M. Khursigara, Karine Thivierge, Melanie K. B. Wills, Roger C. Levesque

**Affiliations:** 1 Institut de Biologie Intégrative et des Systèmes (IBIS), Université Laval, Québec City, Québec, Canada; 2 Faculté de médecine, Université Laval, Québec City, Québec, Canada; 3 G. Magnotta Lyme Disease Research Lab, Department of Molecular and Cellular Biology, University of Guelph, Guelph, Canada; 4 Department of Molecular and Cellular Biology, University of Guelph, Guelph, Canada; 5 Laboratoire de santé publique du Québec, Québec, Canada; 6 Institute of Parasitologie, McGill University, Sainte-Anne-de-Bellevue, Canada

**Keywords:** *Borrelia*, comparative genomics, genome assembly, hybrid assembly, long reads, synteny

## Abstract

The main agent of Lyme borreliosis (LB) in North America, the bacteria *Borrelia burgdorferi*, is spreading in Canada following the northward expansion of its primary tick vectors, *Ixodes scapularis* and *Ixodes pacificus*. Despite the importance of this pathogen for human health, the precise geographical origin and genome structure of Canadian *B. burgdorferi* strains remain to be determined, and no complete genome sequence from this region is available. The complex genome structure of *Borrelia* species makes their assembly challenging, but the latest long-read sequencing technologies and bioinformatics software now enable *de novo* assembly of *Borrelia* genomes with high efficacy. In this study, we sequenced and assembled the genomes of six Canadian *B. burgdorferi* strains to compare their content and structure to additional *Borrelia* genomes from the USA and Europe. We successfully reconstructed the genome, comprising chromosomes and plasmids of the six Canadian strains. These genomes showed an overall similar structure compared to other *B. burgdorferi* strains. Phylogenetic inferences highlighted topological differences in the placement of *B. burgdorferi* strains between the chromosome and the cp26 and lp54 plasmids. Synteny analyses revealed important replicon sequence conservation across strains while highlighting a high proportion of shared gene sequences among the replicons of the same strain, especially for cp32 plasmids. We describe the first complete genomes of Canadian *B. burgdorferi* strains and present a strategy for the assembly, annotation, comparative analysis of plasmids and their evolution in the same bacterial genus. While the genome content and structure of Canadian strains are similar to other *B. burgdorferi* strains, the information in the plasmids and genes they harbour will be useful to elucidate the origins and evolution of LB in Canada.

## Data Summary

The genomes assembled during this study are available in the NCBI Genome database under BioProject PRJNA1130942 and BioSample SAMN42233977 to SAMN42233989 (https://ncbi.nlm.nih.gov/bioproject/PRJNA1130942). The code for the bioinformatics workflow used to assemble the genomes is available on GitHub (https://github.com/AnthonyPiot91/RBGA).

**Impact Statement**

Lyme disease is an emerging public health concern in Canada as its primary bacterial agent, *Borrelia burgdorferi*, spreads northward with its tick vectors. However, little is known about the genetic makeup and evolutionary history of Canadian *B. burgdorferi* strains, limiting our understanding of the pathogen’s origin and adaptation in its new environment. This study presents the first complete genomes of six Canadian *B. burgdorferi* strains, revealing their close genetic relationship to other *B. burgdorferi* strains while highlighting differences in plasmid content and structure. By providing valuable genomic resources, these findings will help trace the origins and evolution of Lyme disease in Canada, ultimately informing public health strategies and disease management.

## Introduction

### Lyme borreliosis

Lyme borreliosis (LB or Lyme disease) is the most common vector-borne disease in temperate regions of the Northern Hemisphere. It has become an emerging disease in Canada due to the range expansion of its main vectors in North America, the black-legged tick, *Ixodes scapularis* [1,3], and the western black-legged tick, *Ixodes pacificus* [4,5]. Following increasing temperatures due to climate change, these ticks colonized new regions in southern Canada from the USA. Consequently, the reported number of LB cases in Canada has increased from 144 in 2009 to 2,525 in 2022 [6]. In North America, the main agent of LB is the bacterium *Borrelia burgdorferi*. Recent phylogenetic studies showed that Canadian *B. burgdorferi* strains do not form a monophyletic clade, nor do they consistently cluster with northern USA strains [7,8]. Consequently, the geographical origin of these invading Canadian strains appears to be diverse and mostly unresolved.

Closely related species form the Lyme borreliosis-associated (LBA) group (also referred to as the *B. burgdorferi sensu lato* complex), currently comprised of 20 named species, including the agents of LB in North America (*B. burgdorferi* and *Borrelia mayonii*) and Europe (*Borrelia garinii* and *Borrelia afzelii*), and non-pathogenic species, including *Borrelia kurtenbachii* [9,11]. More distantly related species responsible for relapsing fever (RF), such as *Borrelia hermsii* also found in North America, form the relapsing fever-associated (RFA) group [12]. A debate currently exists concerning the separation of the *Borreliaceae* family into two genera [9,10, 13,18]. However, in this article, we will refer to the LBA and RFA groups to distinguish between *Ixodes*-transmitted borreliae of the *B. burgdorferi sensu lato* complex and the *Argasidae*-transmitted RFA borreliae, respectively.

Within the LBA group, the different species and strains have various levels of virulence and associated symptoms [19,23]. While some species, such as *B. burgdorferi*, can cause LB, not all strains of the same species result in human disease. The genomic factors responsible for differences in species and strain pathogenicity are poorly understood. However, evidence suggests that some genes involved in pathogenicity and infection of host and zoonotic vectors are present on plasmids [24,25].

### Genome structure

*Borrelia* species are characterized by small (~1.5 Mb) but highly segmented genomes composed of a linear chromosome (~900 Kb), in addition to linear and circular plasmids (up to 21 in *B. burgdorferi* B31) [26,29]. The number and identity of plasmids within *Borrelia* genomes are highly variable between species and even differ between strains of the same species [28,30]. Plasmid identity is primarily based on sequence homology between members of paralogous gene families involved in plasmid maintenance [31]. Members of the Pfam32 protein family, especially, are used in plasmid nomenclature for the different *Borrelia* species based on the homology to the sequences of reference species. *Borrelia* genomes have a dynamic evolution characterized by plasmid loss and gain, replicon rearrangements and plasmid fusion, resulting in a complex mosaic structure [30]. The linear chromosome and plasmids in *Borrelia* genomes are dsDNA molecules with hairpin telomeres [32,33]. These telomeres are composed of almost perfect inverted repeats wrapping around the end of the replicon, forming covalently closed ends. Among the circular plasmids, replicons of the cp32 family appear to be prophage-derived and display high similarity throughout their sequences [33,35]. Many homologous regions are found across and within replicons, significantly increasing genome complexity. The highly variable plasmid content of *Borrelia* species also means that gene content varies greatly between species and strains. *Borrelia* species have minimal metabolic capabilities and encode few biosynthetic pathways [34]. These particularities in replicon and gene content of *Borrelia* genomes reflect their highly specialized, host-associated lifestyle. Therefore, the availability of complete *Borrelia* genomes (i.e. including the chromosome and the full set of plasmids) for comparative genomics, evolutionary history and genomic epidemiology studies is invaluable [35,36].

### Genome assembly

*Borrelia* genomes display an important genome content and structure variability between closely related species or even between different strains of the same species. In addition, genome heterogeneity can occur, where cells of the same strain or from the same isolated culture might have different genome content and structure [36,37]. These rapidly evolving genomes make the use of reference-based genome assembly suboptimal to reconstruct entire *Borrelia* genomes and promote *de novo* assembly. Moreover, the complexity of *Borrelia* genomes increases the difficulty of reconstructing all replicons entirely and accurately in these organisms. Using short-read sequencing, previous studies have reconstructed the chromosome with high confidence but not the plasmids [7].

The advent of new sequencing technologies based on long reads brings new opportunities to reconstruct *Borrelia* genomes more efficiently [38]. By spanning longer regions of the genomes, long reads can resolve repetitive and homologous sequences across replicons. However, long-read technologies typically have lower base calling accuracy than those using short reads, resulting in reads with a higher error rate. Sequencing genomes with both short- and long-read technologies offers the advantage of short reads’ low error rate and long reads’ greater length [36]. This approach can now be used to reconstruct genomes of Canadian *B. burgdorferi* strains, including plasmids.

### Objectives

In this study, we sequence and assemble the complete genomes of six Canadian *B. burgdorferi* strains and seven additional strains belonging to five *Borrelia* species from the USA and Europe with short- and Oxford Nanopore Technologies (ONT) long-read technologies. We then compared the replicon content of Canadian strains to other *Borrelia* isolates and identified syntenic regions within and between genomes. Finally, phylogenetic inferences using marker sets from different replicons were used to better understand the origin and evolution of *B. burgdorferi* strains in Canada.

## Methods

### *Borrelia* strains

#### Strain origin

A total of 13 strains from different *Borrelia* species were sequenced for this project, including six Canadian strains (Table 1). Five Canadian *B. burgdorferi* strains, Bb16-15-2, Bb16-23-2, Bb16-134, Bb16-150 and Bb16-183 [7], were obtained from Canada’s National Microbiology Laboratory (NML). Six strains, two *B. burgdorferi* (B31 and 297), one *B. kurtenbachii* (25015), one *B. afzelii* (BO23), one *B. garinii* (CIP103362) and one *B. hermsii* (HSI serotype 26), were obtained from the American Type Culture Collection (ATCC). These six strains were chosen among available strains in type strain collections that we successfully grew in laboratory. The objective was to validate our assembly pipeline by resequencing reference strains from different *Borrelia* species. *B. burgdorferi* strain (P1286) was generously provided to C.M.K. by Juan Salazar and Kelly Hawley and corresponds to a GFP-expressing B31 strain derivative with a modified cp26 plasmid.

**Table 1.** Information from the sequenced strains in this study

Genus	Species	Strain	Provider	Country	State	Biological origin
*Borrelia*	*burgdorferi*	B31	ATCC	USA	New York State	*Ixodes dammini*
*Borrelia*	*burgdorferi*	297	ATCC	USA	Connecticut	Human cerebrospinal fluid
*Borrelia*	*afzelii*	B023	ATCC	Germany	na	Human skin
*Borrelia*	*garinii*	CIP103362	ATCC	France	na	*Ixodes ricinus*
*Borrelia*	*hermsii*	HSIsero26	ATCC	USA	Washington State	*Ornithodoros hermsi* tick
*Borrelia*	*kurtenbachii*	25015	ATCC	USA	New York State	*Ixodes dammini*
*Borrelia*	*burgdorferi*	GMT-0122	GML	Canada	Ontario	*I. scapularis* (human attached)
*Borrelia*	*burgdorferi*	P1286	*	*	*	*
*Borrelia*	*burgdorferi*	Bb16-15-2	NML	Canada	Manitoba	*I. scapularis*
*Borrelia*	*burgdorferi*	Bb16-23-2	NML	Canada	Manitoba	*I. scapularis*
*Borrelia*	*burgdorferi*	Bb16-134	NML	Canada	NorthWest Ontario	*I. scapularis*
*Borrelia*	*burgdorferi*	Bb16-150	NML	Canada	Nova Scotia	*I. scapularis*
*Borrelia*	*burgdorferi*	Bb16-183	NML	Canada	Nova Scotia	*I. scapularis*

*P1286 is a genetically engineered strain also known as B31 5A4 NP1 GFP. It was provided by Juan Salazar and Kelley Hawley (Connecticut Children’s Medical Centre, Division of Infectious Diseases) to Cezar M. Khursigara [79].

Finally, one Canadian *B. burgdorferi* strain (GMT-0122) was cultivated from a tick by the G. Magnotta lab. The tick was discovered on an adult human male host in Ontario in November 2022 and found to be *Borrelia*-positive by nested PCR targeting the 23S rRNA gene. Half of the bisected tick was immersed in a sterile 1.5 ml microtube containing BSK-H media (Dalynn, BB83-500 or Sigma B8291-500ml) and three antimicrobials (rifampicin, phosphomycin and amphotericin B) and homogenized using a sterile pestle. The inoculated tube was stored at room temperature for 5 days and then placed in a static incubator at 37 °C/5% CO_2_ in G. Magnotta Lab. Briefly, 3 weeks after inoculation, the contents were subcultured into fresh BSK-H with the three aforementioned antibiotics, as well as nalidixic acid to further inhibit the growth of unrelated tick microbiota.

#### Strain culture

*Borrelia* cultures were started from thawed glycerol stocks added to 10 ml aliquots of BSK-H in 15 ml sterile, conical tubes to a final dilution of 1/200. Cultures were grown at 37 °C/5% CO2 without shaking for 5–10 days and cells were quantified using C-Chip hemocytometer slides (DHC-N01, INCYTO) by phase contrast at an objective magnification of 60×. Cells were pelleted in the growth tubes using a swinging bucket rotor at 4.1k ***g*** for 15 min at 4 °C. Cell pellets were resuspended in 500 µl of PBS and pelleted again at 17,100 ***g*** for 5 min at 4 °C in a refrigerated microcentrifuge. Final cell pellets were stored at −80 °C until DNA isolation was performed.

### Library preparation and sequencing

DNA was extracted from pellets using the DNeasy Blood and Tissue Kit from Qiagen following the manufacturer’s recommended protocol for Gram-negative bacteria.

#### Long-read sequencing

The 13 *Borrelia* strains analysed in this study were sequenced on two sequencing runs. The first run included the five strains from NML (Bb16 strains), and the second run included the eight remaining strains. Libraries were prepared using the ligation kit SQK-LSK109 associated with the native barcoding kit NBD-104 from ONT. Long-read libraries were sequenced on an Oxford Nanopore GridION. The five strains from NML were loaded on an R10 flowcell (FLO-11MIN111), and the eight strains from ATCC were loaded on an R9 flowcell (FLO-MIN106). All strains were base called using *guppy v6.4.6* (https://nanoporetech.com/document/Guppy-protocol).

#### Short-read sequencing

Whole-genome shotgun library preparation and sequencing were performed at the Plateforme d’Analyses Génomiques of the Institut de Biologie Intégrative et des Systèmes (IBIS, Université Laval, Québec, Canada). Briefly, 100 ng of genomic DNA was fragmented with a Covaris M220 instrument to produce 600 bp average-size fragments. Whole-genome shotgun barcoded libraries were prepared using 1/5 reaction volumes of the NEBNext Ultra II DNA Library Prep Kit following manufacturer’s instructions. A Beckman Coulter ECHO 525 acoustic liquid handler was used to precisely distribute the reagents. The barcoded libraries were mixed with other samples in a ratio calculated to obtain at least 25X coverage per sample and sequenced for paired-end 300 bp reads on an Illumina Miseq platform using a v3 600 cycles run for the five strains from NML and an Element Biosciences AVITI using a PE300 medium output sequencing kit for the eight remaining strains (six strains from ATCC and two strains from private sources).

### Assembly workflow

**Fig. 1.**
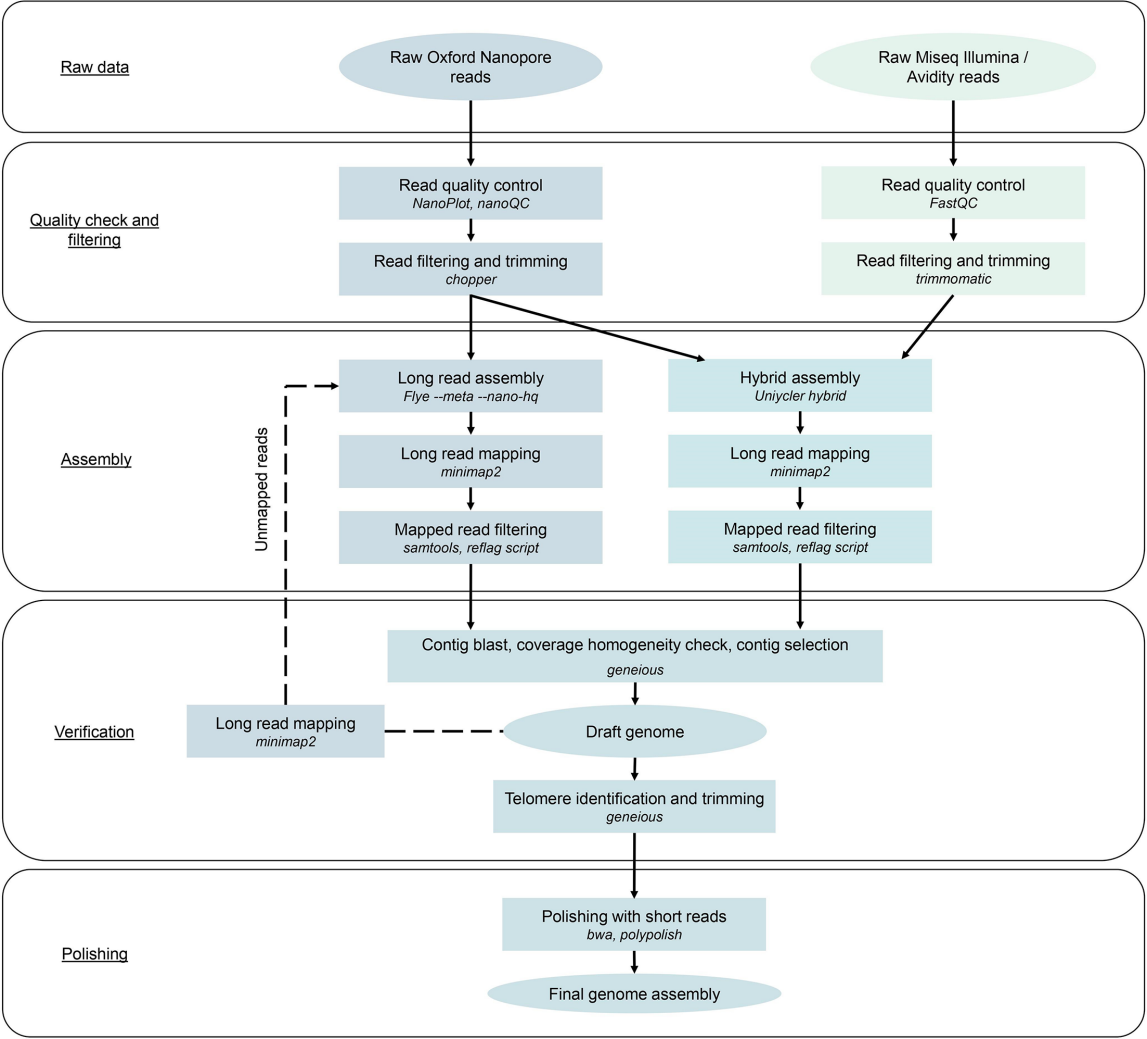
Bioinformatic workflow used to reconstruct the *Borrelia* genomes. This workflow takes advantage of Oxford Nanopore long reads in combination with short reads. First, reads go through quality control, filtering and trimming steps. After cleaning, a long-read-only assembly is conducted with *Flye* and a hybrid assembly with *Unicycler hybrid*. Long reads are then mapped onto the resulting assemblies and filtered for best mapping quality. Next, contig identification relative to *Borrelia* replicons and coverage homogeneity check is conducted to select good-quality contigs that will form the draft genome. Telomeres are then identified and contigs are trimmed to reflect the true replicon sequences. Finally, short reads are used to polish the assembly and correct errors in the nucleotide sequence.

We devised a bioinformatics pipeline to assemble the genomes of different *Borrelia* strains, including the chromosome and the linear and circular plasmids from long and short sequencing reads (Fig. 1). First, the length and quality of raw Oxford Nanopore reads were assessed using *NanoPlot v1.32.1* [39] and *nanoQC v0.9.4* [40]. Using *Chopper v0.3.0*, raw reads were filtered for a minimal length of 2,000 bp and a minimal Phred quality score of 15 and trimmed of the first and last 25 bp [39]. Trimmed Oxford Nanopore reads were used to perform long-read-only assembly using *Flye v2.9.2* in high quality nanopore reads (*--nano-hq*) and metagenome assembly (*--meta*) modes, allowing different coverage between reconstructed contigs to account for differences in plasmid copy number [41,42]. In addition, a read-error rate of 0.032 (*--read-error 0.032*) and two rounds of polishing (*--i 2*) were specified as parameters.

In parallel, short-read quality was assessed using *FastQC v0.12.1* [43]. Raw short reads were filtered for a minimal length of 275 bp and minimal Phred quality score of 30, and adapters were removed using *Trimmomatic v0.39* [44]. Hybrid assembly from trimmed long and short reads was performed using *Unicycler v0.4.8* hybrid mode, where a short-read assembly is first performed and the resulting contigs are bridged using long reads [45].

For verification purposes, trimmed long reads were then mapped on the long read and hybrid assemblies using *Minimap2 v2.24* [46] with the ‘*asm10*’ preset, allowing up to 10% sequence divergence. From the long read and hybrid assemblies, we selected only good-quality contigs defined as contigs: longer than 2,000 bp, with a homogeneous read coverage, and representing a complete replicon sequence, excluding the telomeres for linear replicons. Selected contigs were compared between the long read and hybrid assemblies, and unique contigs were included in the draft genome. Long reads were then mapped onto the draft genome with *Minimap2*, and unmapped reads were used in an additional long-read assembly with *Flye* as described above. Unmapped reads were then mapped onto this new long-read-only assembly, visualized in *Geneious*, and good-quality contigs were added to the draft genome. This process was repeated until no new contig of sufficient quality was recovered.

Once all possible contigs were reconstructed, we identified the hairpin telomeres at each end of *Borrelia* linear replicons by locating the inverted repeat sequences using dot plots and trimming the telomeres at the middle of the palindromic sequences. When telomeres were not found in contigs assembled from the assemblers, contig ends were elongated until telomere regions could be recovered. Circular replicons were circularized by finding homologous regions. Finally, complete draft genomes were polished with trimmed short reads using *BWA 0.7.17* and *Polypolish v0.5.0* [47,48]. The code for the pipeline is available on GitHub (https://github.com/AnthonyPiot91/RBGA).

### Annotation

Final genome assemblies were annotated using *Prokka v1.14.16* [49], with annotation mode set on the bacterial kingdom and translation table number 11 corresponding to the bacterial, archaeal and plant plastid codes. Annotation was based on a list of proteins found in six reference *Borrelia* genomes [*B. hermsii* HSI (GCF_001660005.1), *B. afzellii* Pko (GCF_000222835.1), *B. garinii* PBr (GCA_000172275.2), *B. burgdorferi* JD1 (GCF_000166655.1), *B. burgdorferi* B31 (GCF_000008685.2) and *B. burgdorferi* MM1 (GCF_003367295.1)]. These six reference genomes were selected for their close relatedness to the sequenced strains and for containing replicons present in all of them, thereby minimizing the risk of missing genes present in the sequenced strains but absent from the reference genomes.

### Genome completeness

Genome completeness was assessed using three different approaches. The first two, *BUSCO v5.8.0* [50] and *CheckM v1.2.3* [51], were used to assess the genome completeness of 13 sequenced strains by looking for universal *Borrelia* gene markers within the reconstructed genomes. Because these markers must be found across all *Borrelia* species, they are mostly located on the core replicons and do not represent all *Borrelia* replicons. Therefore, we also used a third tool, *Merqury v1.3* [52], to assess genome completeness by identifying k-mers in the trimmed short reads that are also found within the reconstructed genome sequences.

### Average nucleotide identity, read coverage and GC content

Average nucleotide identity (ANI) of the 13 sequenced strains was assessed using *fastANI v1.34* [53]. Pairwise comparisons between the studied strains were computed to yield an ANI matrix across whole genomes. Cleaned short reads were mapped onto the final assemblies to assess the mean genome coverage of each replicon using *bamtocov v2.7.0* [54]. GC content of each replicon was determined using *bioawk v1.0* (https://github.com/lh3/bioawk).

### Plasmid typing

From the final genome assembly of the 13 sequenced strains, 4 plasmid partitioning genes (*Pfam32*, *Pfam49*, *Pfam50* and *Pfam57/62*) were identified across the 13 sequenced strains using an approach by Kuleshov and colleagues [55]. *InterProScan v5.72-103.0* [56] was used to search the Conserved Domain Database (CDD) and Pfam database for the following accession numbers: cd02038 and cd02042 for *Pfam32* within the CDD, PF01672 for *Pfam49*, PF02890 for *Pfam50* and PF02414 for *Pfam57/62* within the Pfam database.

We identified each replicon’s plasmid type based on the sequence of the respective plasmid partitioning *Pfam32* gene. The *Pfam32* sequence of every replicon in the 13 sequenced strains and 6 additional reference *Borrelia* genomes (*B. hermsii* HSI, *B. afzellii* Pko, *B. garinii* PBr, *B. burgdorferi* JD1, *B. burgdorferi* B31 and *B. burgdorferi* MM1) was aligned using *MAFFT v7.490* [57]. A maximum likelihood phylogenetic tree was then inferred from the alignment using *FastTree v2.1.12* and rooted using midpoint rooting [58]. The plasmid type of every replicon was determined according to the clustering of *Pfam32* sequences within the phylogenetic tree with the reference genomes’ sequences.

### Pangenome

We identified the core genome of *B. burgdorferi* by downloading the complete reference genomes currently available on NCBI genomes for this species. Genomes with less than five replicons are considered incomplete and discarded. Moreover, *B. burgdorferi* B-17 was not included because it lacked the lp54 plasmid universally present in *B. burgdorferi* strains [30]. This left us with 22 complete genomes in addition to the 9 *B. burgdorferi* genomes sequenced in this study (31 genomes total, Table S1, available in the online Supplementary Material). We then used *Panaroo v1.5.2* [59] with default parameters to identify the core genes of this *Borrelia* species.

### Phylogenetic inferences

We reconstructed 4 additional phylogenetic inferences, which included the 31 *B. burgdorferi* genomes used for the pangenome analysis and 4 outgroup *Borrelia* genomes (35 genomes total, Table S1). We originally aimed to reconstruct a whole replicon phylogenetic inference from the chromosome, lp54, cp26 and lp17, as these are found across the LB strains. However, because of the high degree of genomic rearrangements in the lp17 plasmid, it was not possible to produce a sensible alignment for this replicon, and it was excluded from the phylogenetic analyses. Another phylogeny was reconstructed from the commonly used multilocus sequence type (MLST) chromosomal markers [60], including eight housekeeping genes (*clpA*, *clpX*, *nifS*, *pepX*, *pyrG*, *recG*, *rplB* and *uvrA*).

The eight MLST markers and whole replicon sequences for the chromosome and the lp54 and cp26 plasmids were aligned with *MAFFT* v*7.490* [57]. Recombinant regions were masked off the three whole replicon alignments using *Gubbins v3.4.1* [61] with the ‘*extensive search*’ parameter. A Pairwise Homology Index test was then performed with *Phipack v1.1* (https://www.maths.otago.ac.nz/~dbryant/software.html) to verify that recombinant regions had been masked from the alignment. Alignments were then cleaned with *Trimal v1.5.0* [62] using the ‘*gappy-out*’ mode to remove regions with gaps in most sequences. Finally, the best nucleotide substitution models for each alignment were determined using the *MixtureFinder* programme in *IQ-TREE v3.0.1* [63] with the AICc selection criterion. A phylogenetic tree was inferred with *IQ-TREE* based on the best nucleotide substitution models and branch support was tested using 1,000 Ultra Fast Bootstraps.

### Strain typing

MLST and OspC types were determined for the 31 *B. burgdorferi* strains used for the pangenome and phylogenetic analyses. MLST was determined using the pubMLST website (pubmlst.org [64]). OspC gene sequences of the 31 studied strains were aligned with 32 reference OspC sequences from [65] using *MAFFT* v*7.490* [57]. Clustering with OspC reference sequences in a pairwise distance matrix using a threshold of less than 2% sequence divergence was then used to determine the OspC types of the 31 studied strains.

### Synteny

Protein sequences from *Prokka* annotated genes were used to identify syntenic regions within the 13 sequenced *Borrelia* genomes. Homologous regions of collinear genes within strains were identified based on protein sequence best reciprocal hits using *MCScanX v1.0.0* [66]. Syntenic regions within strains were visualized using the *R* package *circlize v0.4.15* [67].

In addition, syntenic regions were identified between the nine *B. burgdorferi* strains sequenced in this study and four additional *B. burgdorferi* strains selected from the inferred chromosome phylogeny to form a representative sampling of the phylogenetic diversity of this species. Synteny between the strains and their different replicons was identified using the *R* package *GENESPACE v1.3.1* [68], which relies on *OrthoFinder* [69] to identify orthologs between strains and use them as homologous regions in the synteny analysis. This approach requires a reference genome to be compared to all other genomes in the analysis. We used *B. burgdorferi* P1286 as a reference because of its high number of plasmids, large genome size and similarity to the B31 reference strain.

## Results

### Genome assembly

The assembly statistics of the 13 sequenced strains can be found in Table 2. In several instances, we observed that the reconstructed contigs from *Flye* were a concatenation of two different plasmids. In these cases, if each part of the contig represented a good-quality contig (see section Assembly workflow), concatenated plasmids were separated and selected to be included in the draft genome. *Flye* could not correctly reconstruct most cp32 plasmids in the studied strains. However, a short-read-first hybrid assembly using *Unicycler hybrid* mode could reconstruct these replicons correctly. The greater the median length of the ONT sequencing reads, the easier it was to identify telomeres because contigs extended further over each end of the telomere. When the ONT read median length was shorter, however, contigs did not always contain the telomeres. Contig extension using reads overlapping the end of the telomere was therefore used until the telomere sequence was recovered. Despite elongation of contigs obtained with *Flye* and *Unicycler hybrid*, we were unable to identify a telomere on one end of lp25 in *B. burgdorferi* Bb16-15-2. Interestingly, we did not recover the telomere in all replicons of *B. hermsii* HSI-sero26 except for one end of lpB58.

**Table 2.** Assembly statistics of the 13 sequence strains

Strain	Total length	Contig no.	Longest	Shortest	*N* count	Gaps	N50	N50*n*
*B. afzelii* B023	1,264,381	13	914,631	9,011	0	0	914,631	1
*B. burgdorferi* 297	1,342,185	16	928,233	11,592	0	0	928,233	1
*B. burgdorferi* B31	1,231,846	12	919,229	12,448	0	0	919,229	1
*B. burgdorferi* Bb16-134	1,337,679	16	913,982	5,325	0	0	913,982	1
*B. burgdorferi* Bb16-150	1,388,621	18	914,082	8,832	4	3	914,082	1
*B. burgdorferi* Bb16-15-2	1,401,150	18	921,424	8,816	1	1	921,424	1
*B. burgdorferi* Bb16-183	1,408,889	18	920,840	15,070	1	1	920,840	1
*B. burgdorferi* Bb16-23-2	1,415,378	19	921,455	4,813	0	0	921,455	1
*B. burgdorferi* GMT-0122	1,305,281	14	920,098	18,131	0	0	920,098	1
*B. burgdorferi* P1286	1,435,686	17	918,978	17,142	0	0	918,978	1
*B. garinii* 103362	1,213,947	10	914,353	21,154	0	0	914,353	1
*B. hermsii* HSI-sero26	1,459,555	11	937,537	11,824	0	0	937,537	1
*B. kurtenbachii* 25015	1,264,094	15	909,021	9,618	0	0	909,021	1

*N* count: number of *N*s (undetermined nucleotides) in the sequences. N50: 50% of the entire assembly is contained in contigs longer than or equal to this value. N50*n*: number of the longest contigs in which 50% of the entire assembly is present.

### Genome statistics and annotation

Genome completeness was very high (>99%) for all 13 sequenced strains according to the universal markers used by *BUSCO* and *CheckM* (Table 3). *Merqury* genome completeness ranged from 96.12 to 99.98%, with *B. burgdorferi* GMT-0122 having the lowest completeness score, whereas the five other Canadian strains had completeness higher than 99.5%. The number of annotated genes in the 13 sequence strains ranged from 1,206 to 1,438 (Table S2). As expected, the number of genes was positively correlated with strains’ genome sizes. The ANI was very high (>98.4%) among all *B. burgdorferi* strains (Table S3). Each Canadian *B. burgdorferi* strain was most closely related to another Canadian strain forming three high similarity pairs: Bb16-15-2 and Bb16-23-2, Bb16-134 and Bb16-150 and Bb16-183 and GMT-0122. ANI decreased gradually from *B. kurtenbachii* to *B. garinii* to *B. afzelii* and decreased significantly for *B. hermsii* (~77%), the only included species of the RFA group.

**Table 3.** Genome completeness of the 13 *Borrelia* strains sequenced and assembled in this study using three different approaches: *BUSCO*, *CheckM* and *Merqury*

Strain	Completeness (%)		
	*BUSCO*	*CheckM*	*Merqury*
*B. afzelii* BO23	100.0	99.78	99.31
*B. burgdorferi* 297	99.7	100.00	98.92
*B. burgdorferi* B31	99.4	99.96	98.61
*B. burgdorferi* Bb16-134	99.7	100.00	99.59
*B. burgdorferi* Bb16-150	99.7	100.00	99.98
*B. burgdorferi* Bb16-15-2	99.7	99.96	99.89
*B. burgdorferi* Bb16-183	99.7	100.00	99.77
*B. burgdorferi* Bb16-23-2	99.7	99.96	99.64
*B. burgdorferi* GMT-0122	99.7	100.00	96.12
*B. burgdorferi* P1286	99.4	99.96	99.70
*B. garinii* 103362	99.7	99.96	99.84
*B. hermsii* HSI-sero26	99.4	100.00	99.43
*B. kurtenbachii* 25015	100.0	99.96	98.53

### Genome content

**Fig. 2.**
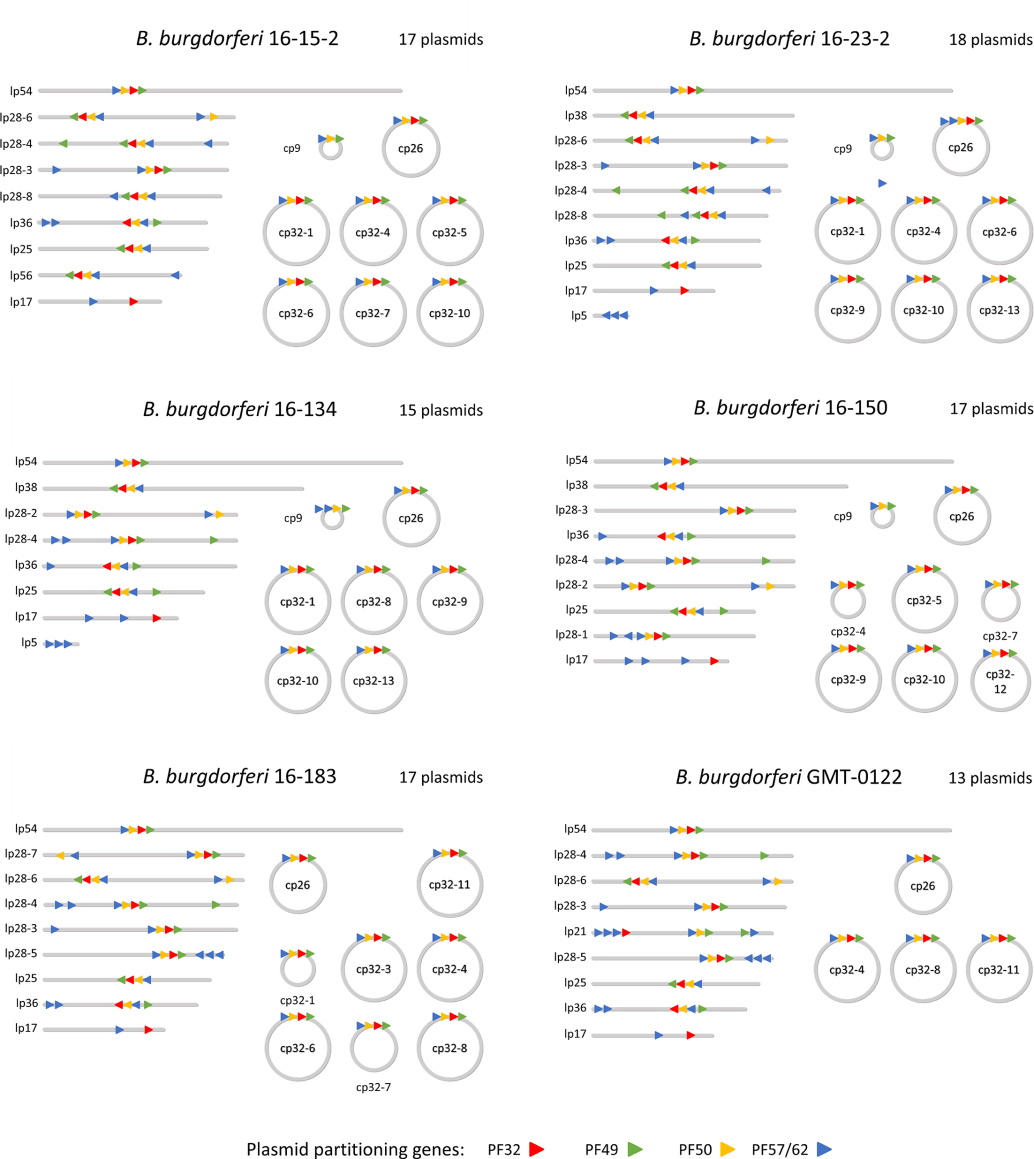
Plasmid content of the six Canadian strains sequenced in this study. Plasmid lengths are to scale. The location and direction of four plasmid partitioning genes are represented on each plasmid.

The plasmid content and location of four Pfam genes in the six Canadian *B. burgdorferi* strains are presented in Fig. 2. The number of reconstructed replicons was 16, 18, 18, 18, 19 and 14 for *B. burgdorferi* Bb16-134, Bb16-150, Bb16-15-2, Bb16-183, Bb16-23-2 and GMT-0122, respectively. For the 13 sequenced strains, total genome size ranged from 1.21 to 1.46 Mbp, which is typical of *Borrelia* species. As a point of comparison, the reference *B. burgdorferi* B31 strain has a total genome size of 1.44 Mbp [26]. The longest replicon, representing the chromosome, was consistently recovered with a size ranging from 909,021 to 937,537 bp. The number of plasmids between strains varied from 9 to 18 (Table S4 and Fig. S2), with the longest recovered plasmid being the megaplasmid in *B. hermsii* HSI-sero26 (182,755 bp) and the shortest plasmid being the lp5 plasmid in *B. burgdorferi* Bb16-23-2 (4,813 bp). Some plasmids were only recovered from one strain. These included all plasmids from *B. hermsii* HSI-sero26 except for cp32-4 and lp32-10 from *B. garinii CIP103362*. Interestingly, 12 replicons were recovered from the *B. burgdorferi* B31 strain sequenced in this study, compared to the 22 replicons in the reference B31 strain. Eleven plasmids (lp56, lp38, cp32-8, cp32-7, cp32-1, cp32-6, lp28-4, lp25, lp21, cp9 and lp5) were lost and one plasmid (cp32-5) gained compared to the B31 reference strain. Sequence similarity between cp32 plasmids within strains was high, with most cp32 pairs having ~80–90% sequence identity.

We determined the core and accessory replicons of the studied *Borrelia* species based on the presence/absence of their replicons across strains (Table S4). The only replicon found in all 13 studied strains was the chromosome. Briefly, 3 replicons (lp17, cp26 and lp54) were found in all 12 strains of the *Borrelia* LBA group and constitute, with the chromosome, the core replicons of these species. In addition, within the LBA group, 2 replicons (cp32-4 and lp36) were found in 11 strains, and 2 replicons (lp28-3 and lp28-4) were found across 10 strains. The six Canadian strains all possessed the six following plasmids: cp26, lp17, lp25, lp28-4, lp36 and lp54 (Table S4).

While chromosome length is relatively stable across strains, plasmid length showed significant variation between strains (Table S5). Circular plasmids cp32-1, cp32-3, cp32-4, cp32-7, cp32-8, cp32-9 and cp32-11 were shortened by half or more in at least one of the 13 sequenced strains. Linear plasmids lp36 and lp56 also displayed a large reduction in length, whereas lp17 and lp28-4 were significantly elongated in at least one strain. Interestingly, the six Canadian strains presented a considerable reduction of lp36. The lp17 plasmid had a highly variable structure, with only half of the plasmid that appears to be conserved across strains. On the contrary, cp26 had a very conserved sequence across strains of the LBA group.

GC content also varied significantly between replicons (Table S6). The chromosome had a relatively stable GC content of ~0.285 across strains. Some plasmids such as cp9, lp5, lp17, lp21 and lp28-5 had significantly lower GC content (<0.25), while other plasmids such as lp28-1, lp28-2, lp28-6, lp28-7 and lp28-8 had consistently higher GC content (>0.31). Noteworthily, most replicons of *B. hermsii* HSI-sero26 had higher levels of GC content (>0.30).

We also examined replicon coverage by mapping trimmed short reads onto the assembled genomes and normalizing values relative to the chromosome coverage for each strain (Table S7). Once again, we found high heterogeneity in the coverage of the different replicons, with coverage ranging from 0.48 to 9.29 times that of the chromosome. Circular plasmid cp32-10 was consistently underrepresented compared to the chromosome (~0.5×), whereas cp9 was consistently largely overrepresented (>3×). Coverage for each replicon was not consistent across strains; some plasmids, such as cp32-4 and cp32-5, were underrepresented relative to the chromosome in some strains and overrepresented in other strains, indicating differences in plasmids’ copy number across strains.

Our pangenome analysis across 31 *B. burgdorferi* complete genomes identified 928 core genes (found in 99–100% of strains), 11 soft genes (95–99%), 736 shell genes (15–95%) and 232 cloud genes (0–15%) from a total of 1,907 genes (Table 4).

**Table 4.** Results from the pangenome analysis performed by Panaroo on 31 *B. burgdorferi* strains

Category	Definition	Gene no.
Core genes	(99%≤strains≤100%)	928
Soft core genes	(95%≤strains<99%)	11
Shell genes	(15%≤strains<95%)	736
Cloud genes	(0%≤strains<15%)	232
Total genes	(0%≤strains≤100%)	1,907

### Phylogenetic inferences

We identified the *Pfam32* gene in all reconstructed replicons except in the lp5, lpG27 and cp9 plasmids. These three plasmids were, therefore, named based on blast similarity to reference genome replicons. Fig. S2 represents a phylogenetic tree of the *Pfam32* gene found across the replicons of the 13 sequenced strains and 6 reference strains used for gene annotation. The Canadian *B. burgdorferi* strains always clustered more closely with *B. burgdorferi* strains from the USA and sometimes with the *B. kurtenbachii* strain, also from the USA. In two occurrences, sequences of different plasmids have been found to cluster together (cp6.5 with lp12 and cp32-10 with lp56). The plasmids cp6.5 and lp12 have almost identical sequences; the sequence similarity between these two replicons could be attributed to a linearization of cp6.5 or a circularization event of lp12. The sequence clustering of cp32-10 and lp56 may be attributable to structural rearrangement events that shuffled the *Pfam32* gene between these replicons.

**Fig. 3.**
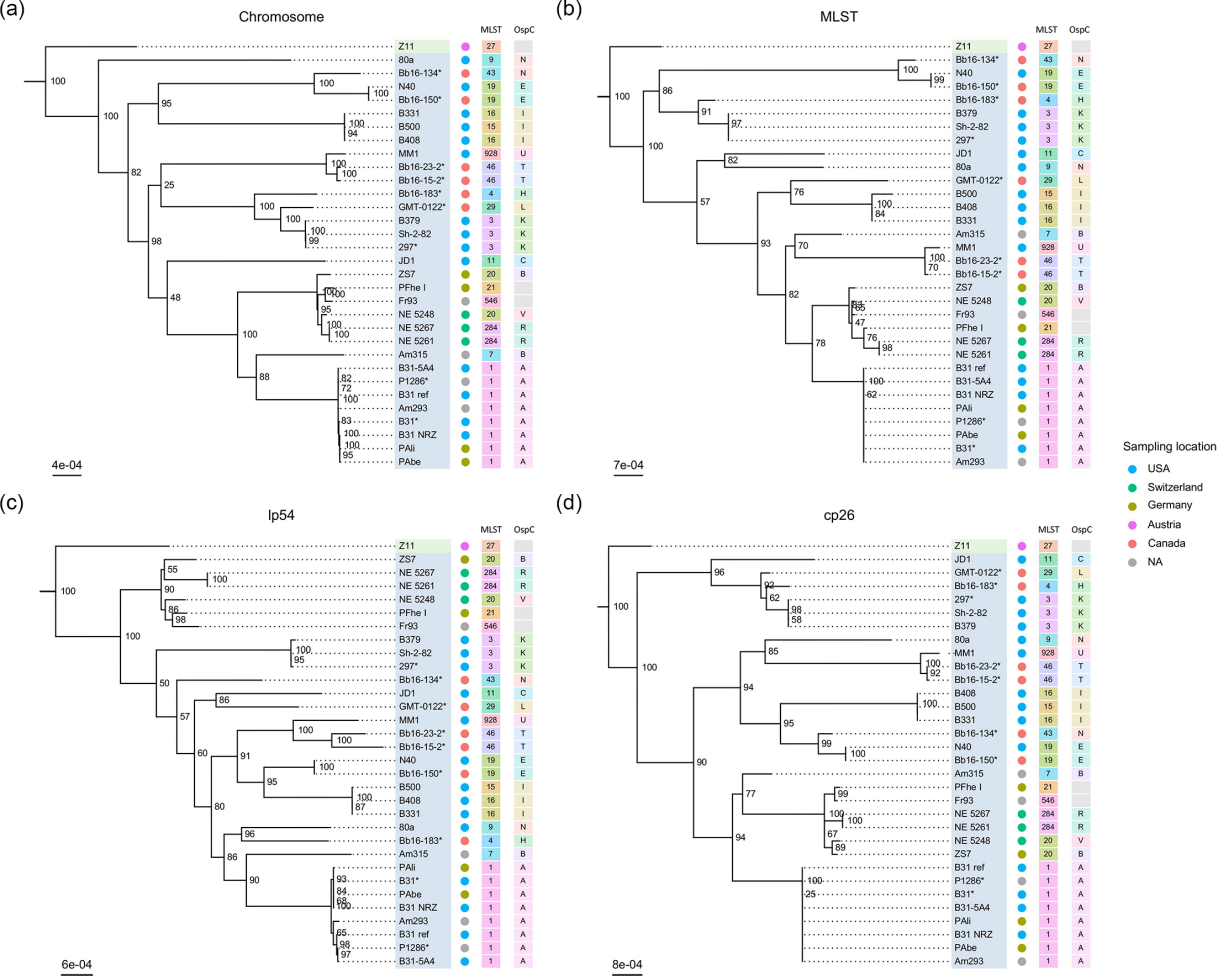
Different tree topologies for 31 *B. burgdorferi* strains obtained from four maximum likelihood phylogenetic inferences based on (**a**) whole chromosome, (**c**) whole lp54, (**d**) whole cp26 sequences and (**b**) eight MLST chromosomal markers. Strains sequenced in this study are indicated by an asterisk. Node labels indicate node Ultra Fast Bootstrap support values. *B. burgdorferi* strains are highlighted in blue and *Borrelia finlandensis* in green. Out of the four outgroup strains used, only the closest to *B. burgdorferi* (*B. finlandensis* Z11) is shown. Strain’s geographic sampling location, MLST and OspC type are plotted against the tree.

We also reconstructed four phylogenetic inferences based on eight chromosomal MLST markers and whole chromosome, lp54 and cp26 sequences (Fig. 3a–d). These phylogenies had overall high node support except for a couple of internal nodes in each tree. Significant topological differences appear between phylogenies. Notably, the placement of *B. burgdorferi* 80a, JD1, Am315, Bb16-183, Bb16-134 and GMT-0122 differed between the four topologies. *Borrelia* strains did not cluster by sampling location. The six Canadian strains did not form a monophyletic group, but *B. burgdorferi* Bb16-23-2 and Bb16-15-2 always formed a monophyletic pair. Bb16-150 always forms a monophyletic clade with N40, with Bb16-134 as a sister strain in the MLST and whole chromosome and cp26 phylogenies. The placement of *B. burgdorferi* Bb16-183 and GMT-0122 was variable. P1286 was always found in the monophyletic clade form by the eight strains most similar to the reference B31 strain.

The 31 studied *B. burgdorferi* strains clustered by MLST and OspC types in the four phylogenies. Bb16-23-2 and Bb16-15-2 belonged to MLST 46 and OspC type T. Bb16-134 belonged to MLST 43 and OspC type N. Bb16-150 belonged to MLST 19 and OspC type E, similar to strain N40. Bb16-183 belonged to MLST 4 and OspC type H. GMT-0122 belonged to MLST 29 and OspC type L. P1286 belonged to MLST 1 and OspC type A, similar to the B31-like strains. OspC types for *B. burgdorferi* Fr93 and PFhe I could not be determined, as they did not cluster closely with any reference OspC accession in our multiple alignment pairwise distance matrix.

### Synteny

**Fig. 4.**
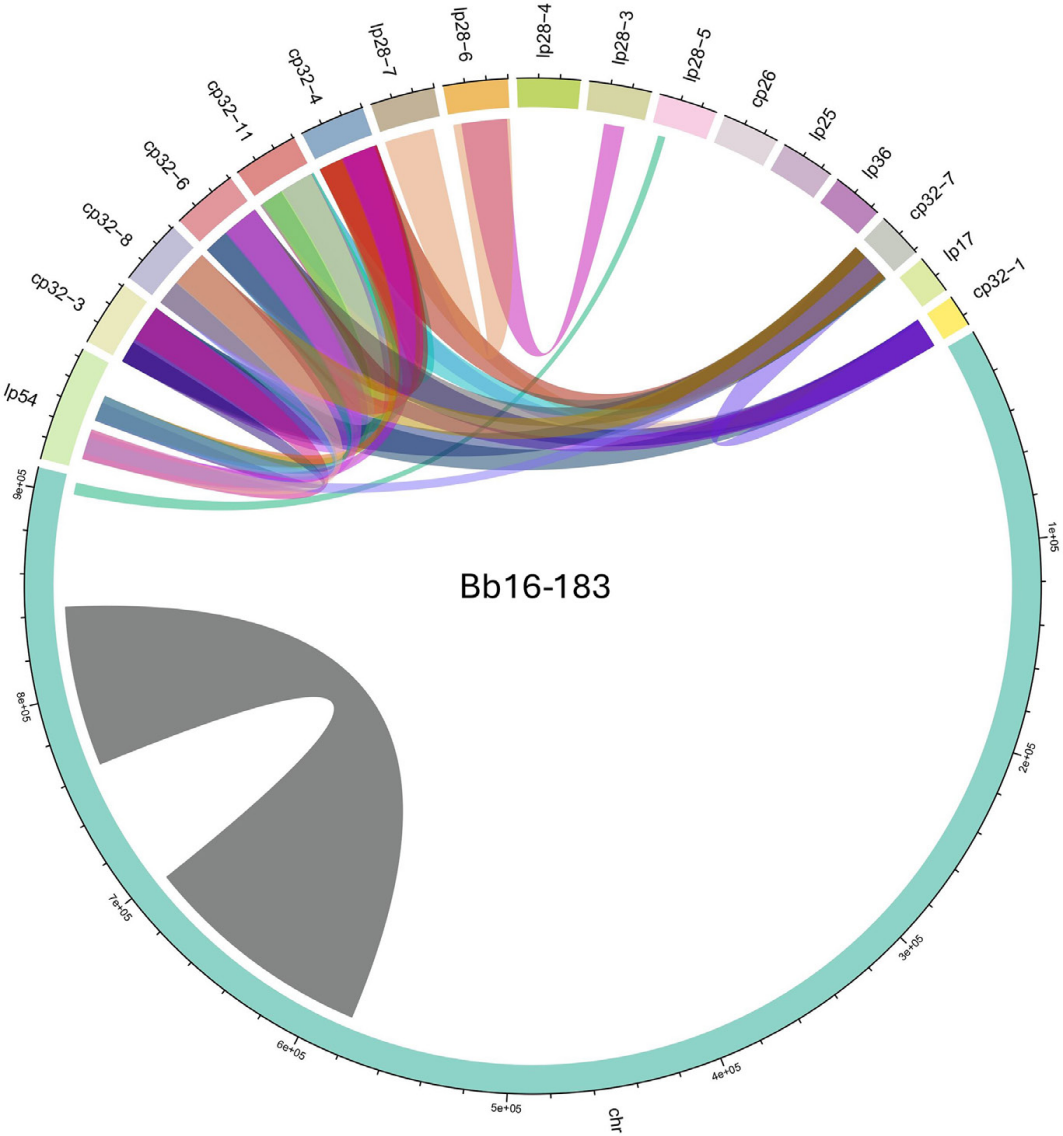
Genome synteny within *B. burgdorferi* Bb16-183 strain. Peripheral segments represent replicons, and ribbons link homologous regions of collinear genes across the genome. Many plasmids share a significant amount of homologous regions in their sequences.

Identifying syntenic regions within the genomes of each of the 13 sequenced *Borrelia* strains highlighted homologous regions between replicons of the same strains (Figs 4 and S3–S5). All strains displayed synteny between two regions within their chromosomes. Within each strain, all cp32 plasmids had high sequence homology between each other. Moreover, two regions of the lp54 plasmid had high sequence similarity with cp32 plasmids. Canadian strains displayed similar syntenic patterns within their genomes compared to the other studied strains, except for a homologous region found in *B. burgdorferi* Bb16-183 and GMT-0122, located at the end of the chromosome and in plasmid lp28-5.

**Fig. 5.**
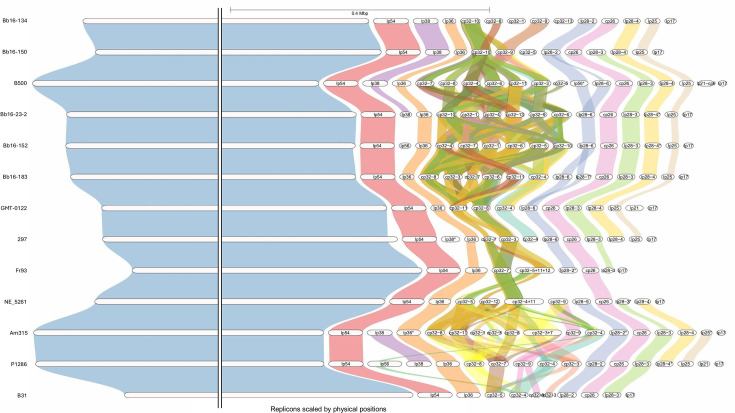
Genome synteny across the 13 *B. burgdorferi* strains. Nine strains were sequenced in this study and four reference strains were used. White horizontal bars represent replicons for each strain genome. Syntenic regions are represented by vertical ribbons between replicons of the different strains. Synteny across the chromosomes of all strains is homogeneous and complete; we, therefore, truncated the chromosome length for visualization purposes. Ribbons are partly transparent and overlapping ribbons lead to new and darker colours, as is the case for synteny between the cp32 plasmids. GENESPACE identifies synteny between strains by comparing a reference genome, in this case P1286, to all other strains. Only replicons with identified synteny with the reference genome are displayed.

Syntenic regions between 13 *B. burgdorferi* strains were also identified (Fig. 5). Sequence conservation was high across strains, with the chromosome and most plasmids showing conserved sequences of collinear genes between strains along their entire sequences. Sequence similarity of different cp32 plasmids between strains was also high, with most cp32 plasmids showing syntenic regions with multiple cp32 plasmids in other strains. Interestingly, lp56 in *B. burgdorferi* P1286 displayed syntenic regions with cp32 plasmids. In addition, lp28-6 plasmids showed high sequence similarity with the lp28-7 plasmid found in strain Bb16-183 and the lp56 plasmid of strain B500. Moreover, lp28-2 plasmid showed high synteny with plasmid lp28-6 and the lp28-9 plasmid of strain NE_5261. The six Canadian *B. burgdorferi* strains showed similar patterns of inter-strain synteny compared to other strains.

Syntenic regions require a minimum number of collinear genes in order to be identified; therefore, a lack of synteny in Fig. 5 does not necessarily mean a total absence of homologous regions between different strains’ replicons but that the number of collinear genes did not meet the threshold of our analysis.

## Discussion

### Hybrid assembly

Currently, using only one type of sequencing reads and one assembler does not allow for the complete and accurate assembly of every replicon within *Borrelia* genomes [28]. In this study, we sequenced *Borrelia* genomes using short reads produced by either Element Biosciences AVITI or Illumina MiSeq and long reads produced by ONT GridION. The assembly pipeline described herein enabled the efficient reconstruction of *Borrelia* chromosomes and plasmids by combining contigs produced by *Flye*, a long-read assembler, and *Unicycler hybrid*, a short-read-first hybrid assembler. Cp32 plasmids were the most challenging replicons to reconstruct. High sequence similarity and within-strain recombination between these plasmids represent a significant challenge for assembly software. However, using short-read-first assembly bridged by long reads implemented by *Unicycler hybrid* enabled the reconstruction of these replicons. We have integrated several quality control steps in our assembly pipeline to prevent misassemblies of the cp32 plasmids: (1) the resequencing of previously assembled reference *Borrelia* strains was a way to validate our method by comparing replicons sequence from resequenced reference strains; (2) a small amount of nanopore sequencing reads corresponded to the full length of the cp32 plasmids and were used to validate our cp32 assemblies; and (3) the mapping of short reads onto long-read-assembled cp32 gave us an additional clue to the quality of the assembly, as homogeneous mapping coverage was indicative of correct assembly. *B. burgdorferi* GMT-0122 had a lower completeness score according to *Merqury* compared to all other sequenced strains. This strain is likely missing at least one plasmid in our assemblies. Unfortunately, we could not improve completeness with the available sequenced data. Telomere identification of linear replicons was also a significant challenge during the assembly of the studied *Borrelia* genomes. Assemblies of ONT libraries with greater read length greatly facilitated the identification of the palindromic telomere regions. Replicon elongation was necessary in cases where the telomere region was not present in contigs produced by the two assemblers. We were unable to identify *B. hermsii* telomeres for linear replicons except lpB58. This is at odds with what has been found in other RF group species, such as *Borrelia miyamotoi*, where telomeres are present on linear replicons [55].

Recently, Hepner and colleagues [70] assessed different strategies for reconstructing the genomes of three *Borrelia* species from long and accurate reads from PacBio HiFi and Illumina MiSeq short reads using three different assemblers. Similarly to the conclusion of our study, they highlighted the importance of a workflow combining multiple assemblers to correctly assemble all linear and circular replicons in *Borrelia* genomes. The long-read sequencing technologies, ONT and PacBio, each have advantages and drawbacks and have successfully been used to reconstruct *Borrelia* genomes [28,55, 70,75]. Prioritizing an adequate coverage of the target genomes and maximizing the length of the sequenced reads appear to be key elements for successfully assembling every replicon in *Borrelia* genomes. In 2018, Tyler and colleagues [7] used whole-genome short-read sequencing to assemble the genomes of 64 *B. burgdorferi* strains from southern Canada, including the five *B. burgdorferi* Bb16 strains used in our study, but plasmid sequences were not fully reconstructed. Nonetheless, the reconstructed chromosome sequences from the five *B. burgdorferi* Bb16s strains from our study and theirs are quite similar, with a sequence identity higher than 99.9%. More recently, Russell and colleagues sequenced and assembled the genomes of 51 western Canadian *Borrelia* strains of the LBA group, also using whole-genome short-read sequencing [8]. The focus of the study was on assessing strain genetic diversity and sequence types and did not emphasize replicon reconstruction. The assemblies presented here, therefore, represent the first complete genome reconstructions (i.e. full chromosome and plasmid sequences) of *B. burgdorferi* isolates sampled in Canada. Such complete assemblies are highly valuable because they allow tracking the evolutionary history of different replicons and the pathogenic elements they may possess.

### *Borrelia* evolutionary history

*Borrelia* strains sampled in different countries in Europe and North America do not cluster by geographic regions. For example, *B. burgdorferi* strains isolated in Canada (Bb16s and GMT-0122) are distributed across the phylogenetic tree for this species, as previously demonstrated by Tyler *et al.* [7]. Only *B. burgdorferi* Bb16-15-2 and Bb16-23-2 strains, both isolated from the same region in Manitoba, Canada, formed a geographically coherent group in our phylogenetic inferences. The two strains (Bb16-150 and Bb16-183) sampled in Nova Scotia, Canada, did not form a monophyletic group, as was the case for the two strains (Bb16-134 and GMT-0122) sampled from Ontario, Canada. Therefore, *B. burgdorferi* strains found in the same Canadian provinces do not have a common geographical origin and appear to have a more complex migration pattern than a simple northward expansion from the northern USA.

The phylogenetic relationships between *B. burgdorferi* strains differed according to the set of markers used. The topology between phylogenies inferred from eight chromosomal MLST markers and whole chromosome sequences differed significantly and indicated that limited gene marker sets are not representative of the full evolutionary history of the bacteria. Topological differences between the different replicons have previously been demonstrated [76]. *Borrelia* chromosomes and plasmids may have distinct evolutionary histories due to horizontal gene transfer mechanisms, such as transduction and recombination, and different mutation and selection rates [77,78].

### Comparative genomics

We found diverse plasmid compositions across the studied *Borrelia* strains [73]. Such variation in plasmid composition prevents reference-based assembly, even when the reference and assembled genomes belong to the same strain [75]. Despite this variation, the *B. burgdorferi* core replicons we identified were similar to the findings of previous studies [70]. The P1286 strain originates from a B31 strain with a modified cp26 plasmid [79]. Therefore, the high sequence similarity between the sequenced *B. burgdorferi* B31 and P1286 strains was expected. These similarities are reflected in the synteny analyses. The six Canadian strains did not display unique plasmid contents compared to other *B. burgdorferi* strains and harboured core replicons composed of the chromosome and six plasmids (cp26, lp17, lp25, lp28-4, lp36 and lp54). However, given the diverse origins of Canadian strains, it is likely that increasing the number of strains will reduce the number of core replicons of *B. burgdorferi* strains sampled in Canada.

Fast changes occur in the genome content and structure of *Borrelia* bacteria, as evidenced by the loss of several plasmids in the *B. burgdorferi* B31 strain sequenced in this study compared to the reference B31 strain [37,80,82]. This rapid evolution of *Borrelia* strains calls for a more precise taxonomic determination of *Borrelia* strains. Indeed, previous studies showed that laboratory culture can lead to plasmid loss [37,82]. A strain isolated several years or even decades ago will very likely show a different genome organization today compared to the time of isolation. This also raises the question of how intact a laboratory-replicated *Borrelia* strain can remain through time. Our *B. burgdorferi* B31 strain also appeared to have ‘gained’ the cp32-5 plasmid compared to the reference B31 strain. A detailed investigation found this plasmid to be an almost exact match for plasmid cp32-5 from *B. burgdorferi* Pali, cp32-5-1 from *B. burgdorferi* PAbe and cp32-5-1 from *B. burgdorferi* B31 NRZ, three very closely related strains also included in this study as reference genomes [83]. Moreover, Casjens and collaborators [84] reported the occurrence of cp32-5 in non-reference B31 strains in 1997 while studying homology of cp32 plasmids in B31 strains of various origins. Therefore, while plasmid cp32-5 was not identified in the original complete genome assembly of strain B31 [26], other closely related strains possess the plasmid. It appears that the B31 strain we have been provided with by the ATCC was not an exact replica of the B31 reference strain but that of another B31 strain that contained the cp32-5 plasmid.

We found that replicon sequences were well conserved across all studied *Borrelia* strains. Given its large size relative to other replicons, the chromosome sequence is especially conserved, as evidenced by the large homologous blocks of collinear genes observed on this replicon across the different *B. burgdorferi* strains. The only specific syntenic feature found in Canadian strains was a homologous region between the end of the chromosome and plasmid lp28-5 in *B. burgdorferi* Bb16-183 and GMT-0122 strains sampled in Nova Scotia and Ontario, respectively.

The strong sequence homology observed between replicons of the same strains, especially in cp32s, was largely responsible for the difficulty in reconstructing some plasmid sequences in assembly workflows. This high proportion of shared blocks of collinear genes in plasmid sequences within the same *Borrelia* strain must lead to an important amount of redundant genetic information. Moreover, the numerous genome rearrangements observed in *Borrelia* species and the mosaic structure of replicon sequences are likely to be important for pathogenicity [30].

## Conclusion

Despite their small sizes, *Borrelia* genomes are challenging to assemble from sequencing reads. However, the latest sequencing technologies and assembly software allow the full reconstruction of *Borrelia* genomes. Combining two assembly software to take advantage of long and short sequencing data, we successfully reconstructed six Canadian *B. burgdorferi* genomes. The availability of the complete set of chromosomes and plasmids enabled comparing plasmid content and structure between *Borrelia* isolates to decipher the origin and evolution of Canadian strains. The diversity of structural rearrangements, sequence redundancy and plasmid composition are essential factors in the genomic epidemiology of *Borrelia*. This study advances the characterization of the genomic landscape of *B. burgdorferi* strains found in Canada and will help to better understand and control the emergence of LB in new regions of North America.

## Supplementary material

10.1099/mgen.0.001606 Uncited Supplementary Material 1.

10.1099/mgen.0.001606 Uncited Supplementary Material 2.

## Abbreviations

ANI: average nucleotide identity
ATCC: American Type Culture Collection
CDD: Conserved Domain Database
LB: Lyme borreliosis
LBA: Lyme borreliosis-associated
MLST: multilocus sequence type
NML: National Microbiology Laboratory
ONT: Oxford Nanopore Technologies
RF: relapsing fever
RFA: relapsing fever-associated
