# Supplementary material for: Unravelling the genomic landscape of Canadian Borrelia burgdorferi: a comparison across global strains
Source: Microb Genom. 2026 Jan 8;12(1):001606. doi: 10.1099/mgen.0.001606 (PMC12813429; doi:10.1099/mgen.0.001606)
Supplement: Uncited Supplementary Material 1. [file mgen-12-01606-s001.pdf]

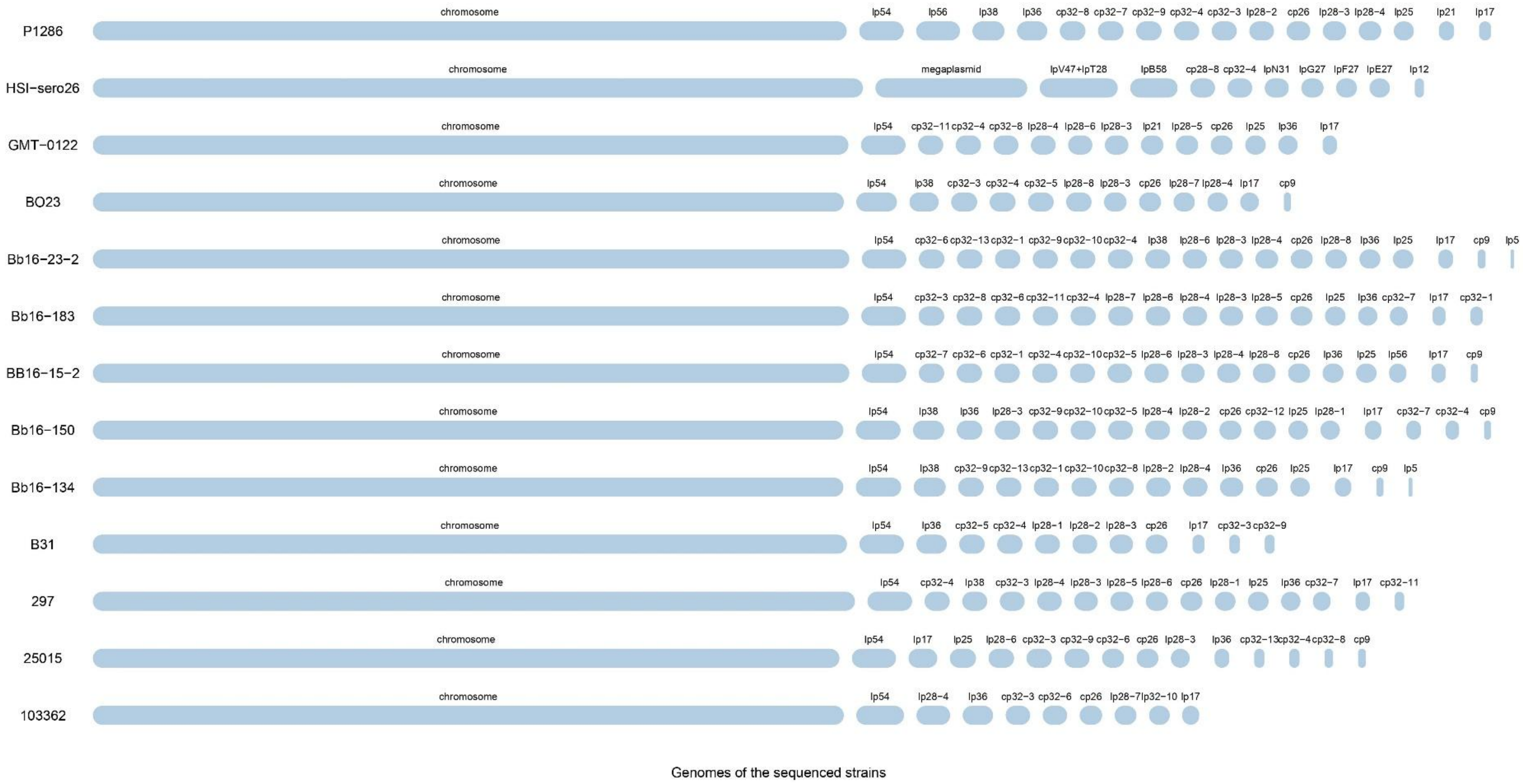

Figure S1. Genome structure of the 13 sequenced Borrelia strains ordered by length.

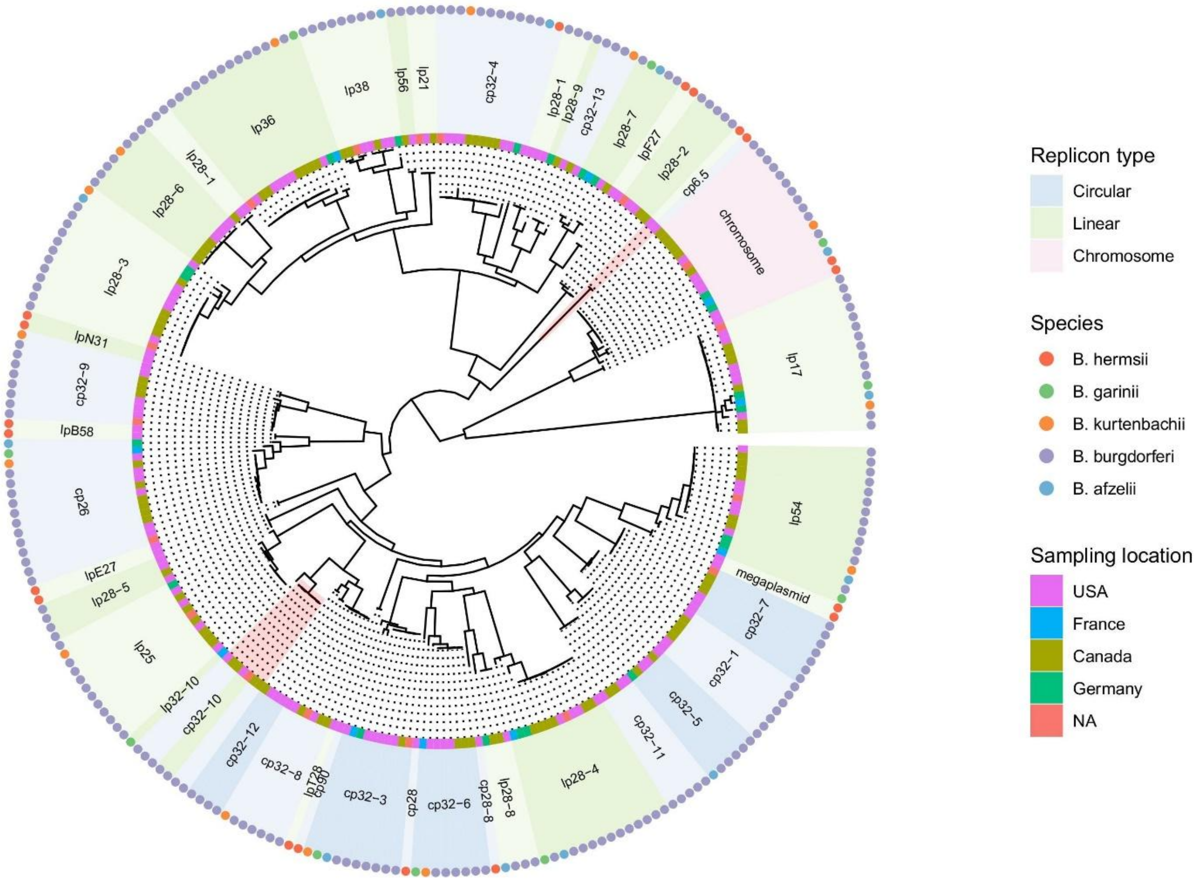

Figure S2. Maximum likelihood phylogenetic tree of the Pfam32 plasmid partitioning genes from 19 Borrelia strains. The rings, from center outward, 1) indicate the sampling location of the strain, 2) the plasmid compatibility group name and type of the replicon on which the PFam32 gene was found, and 3) the five Borrelia species. Red shadings indicate situations where linear and circular plasmids of different plasmid compatibility group cluster together: i) two sequences of lp56 in B. burgdorferi B31 and P1286 are nested within sequences of the cp32-10 plasmid, and ii) the linear plasmid lp12 and circular plasmid cp6.5 plasmid of B. hermsii form a monophyletic group.

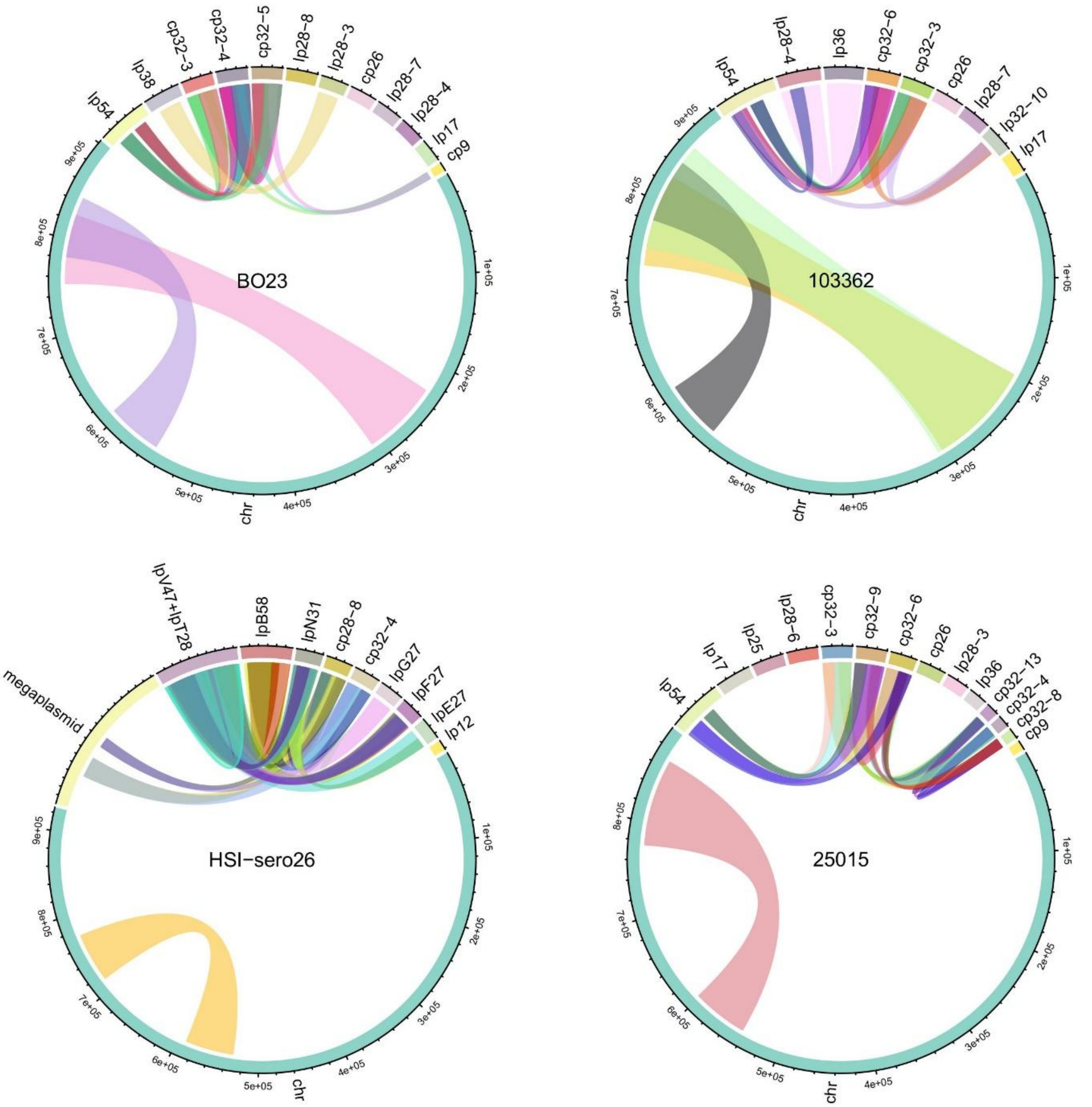

Figure S3. Genome synteny within four Borrelia strains. Circular segments represent replicons and ribbons link homologous regions of collinear genes across the genome.

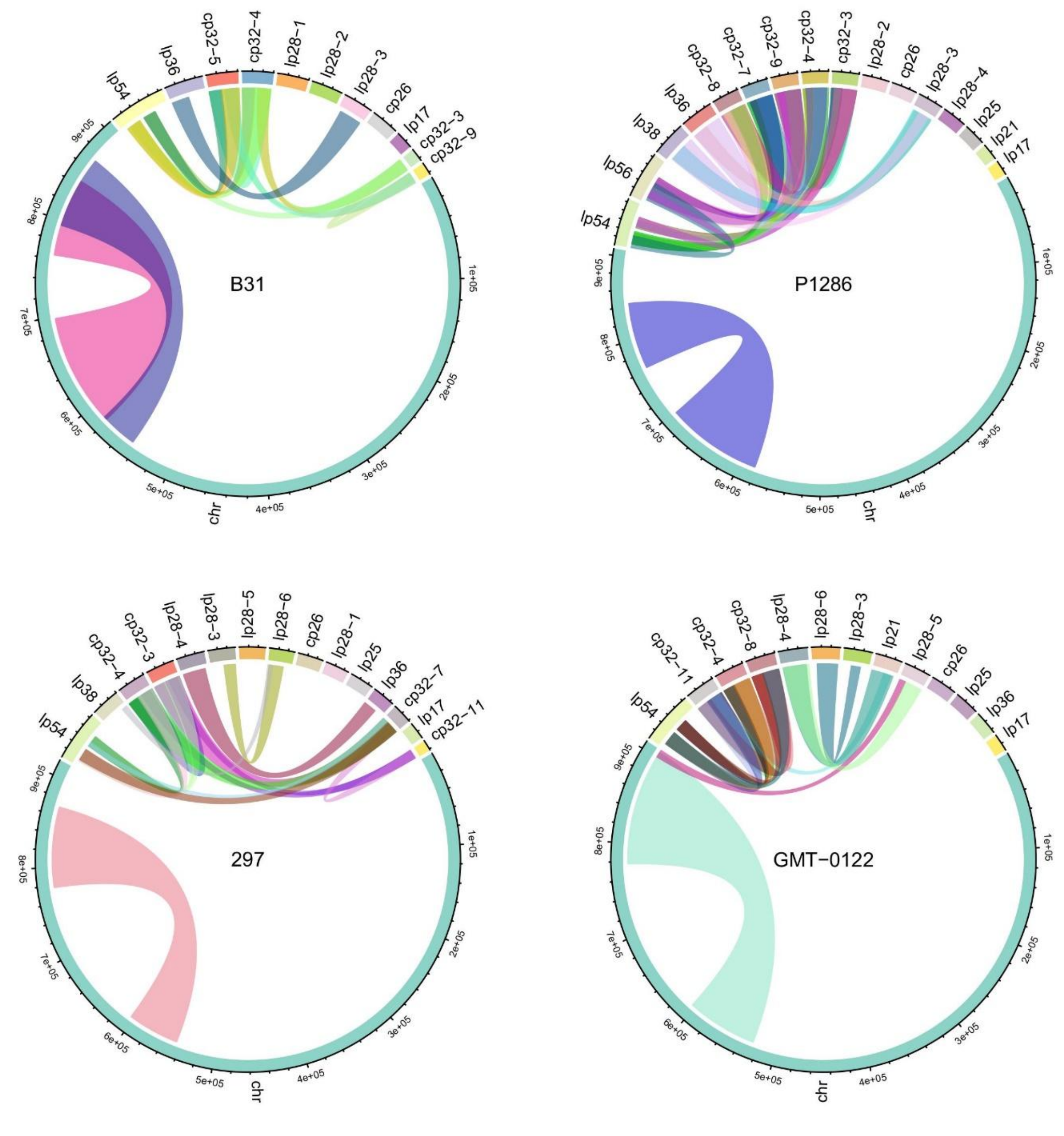

Figure S4. Genome synteny within four Borrelia strains. Circular segments represent replicons and ribbons link homologous regions of collinear genes across the genome.

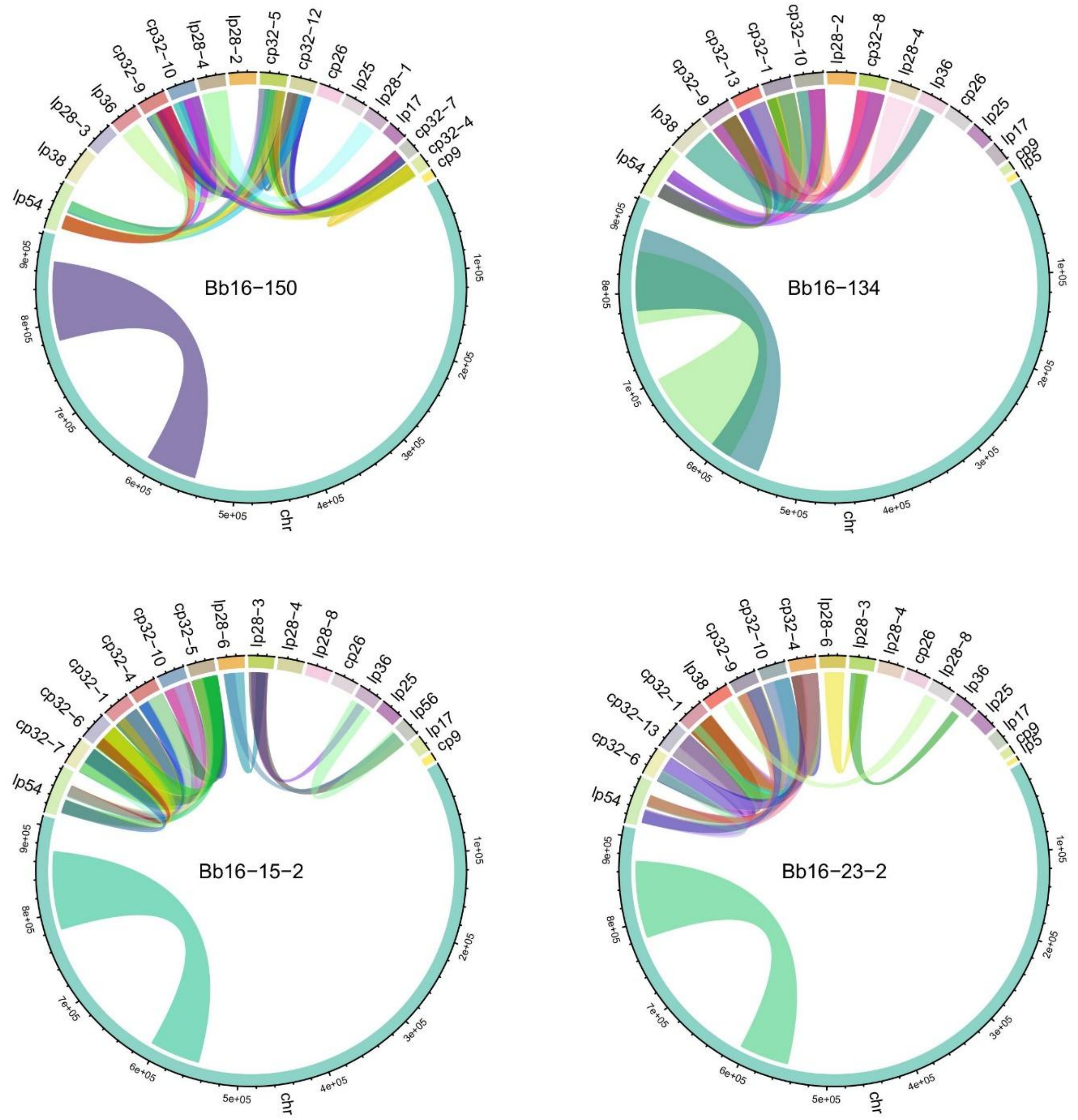

Figure S5. Genome synteny within four Borrelia strains. Circular segments represent replicons and ribbons link homologous regions of collinear genes across the genome.
